# Supplementary material for: Effectiveness and safety of rivaroxaban versus warfarin in Taiwanese patients with end-stage renal disease and nonvalvular atrial fibrillation: A real-world nationwide cohort study
Source: PLoS One. 2021 Apr 8;16(4):e0249940. doi: 10.1371/journal.pone.0249940 (PMC8031437; doi:10.1371/journal.pone.0249940)
Supplement: S1 Table — (DOCX) [file pone.0249940.s001.docx]

**S1 Table. Disease Diagnosis Codes According to ICD-9-CM, ATC Classification of Medications, and Reimbursement Codes for Procedures**

| **Clinical outcomes or comorbidities** | **ICD-9-CM** |
| --- | --- |
| Gastrointestinal bleeding | 456.0, 456.20, 530.7, 530.82, 531.0, 531.2, 531.4, 531.6, 532.0, 532.2, 532.4, 532.6, 533.0, 533.2, 533.4, 533.6, 534.0, 534.2, 534.4, 534.6, 535.01, 535.11, 535.21, 535.31, 535.41, 535.51, 535.61, 537.83, 562.02, 562.03, 562.12, 562.13, 569.3, 569.85, 578.0, 578.1, 578.9 |
| Other non-critical site bleeding | 287.8, 287.9, 599.7, 596.7, 770.3, 784.7, 784.8, 786.3 |
| Intracranial bleeding | 430, 431, 432, 432.0, 432.1, 432.9, 767.0, 852– 852.5, 853–853.1 |
| Other critical site bleeding | 362.81, 363.61, 363.62, 376.32, 379.23, 423.0, 459.0, 568.81, 719.1 |
| Ischemic stroke | 433 –433.9, 434 –434.9, 436 |
| Systemic embolism | 444 – 444.9 |
| Congestive heart failure | 428 – 428.9, 402.11, 402.91, 404.11, 404.13, 404.91, 404.93 |
| Peptic ulcer disease | 531 – 534.9 |
| Hypertension | 401-405 |
| Diabetes | 250 – 250.9 |
| Chronic liver disease | 571.2, 571.5, 571.6, 571.4 – 571.49, 572.2 – 572.8 |
| Hyperlipidemia | 272.0 – 272.4 |
| Chronic obstructive pulmonary disease | 490 – 492, 494, 496 |
| Valvular heart disease | 394 – 397, 424.0 – 424.2, 746.3 – 746.6 |
| Malignancy | 140—165, 170—176, 179–208, 230–234 |
| End-stage renal disease | 585, 403.01, 403.11, 403.91, 404.02, 404.03, 404.12, 404.13, 404.92, 404.93 |
| **Medications** | **ATC code** |
| Nonsteroidal anti-inflammatory drugs | M01AA, M01AB, M01AC, M01AE, M01AG, M01AH, M01AX |
| Glucocorticoids | H02AB |
| Antiplatelet drugs | B01AC04, B01AC05, B01AC06, B01AC07, B01AC23, B01AC24 |
| Proton pump inhibitors | A02BC |
| HMG CoA reductase inhibitors | C10AA |
| Angiotensin-converting-enzyme inhibitors | C09AA, C09BA, C09BB, C09BX |
| Angiotensin II antagonists | C09CA, C09DA, C09DB, C09DX |
| Erythropoiesis-stimulating agents | B03XA01, B03XA02, B03XA03 |
| **Procedures** | **Reimbursement code** |
| Transfusion | 94001C, 93010C, 93011C, 93013C, 93001C, 93002C, 93003C, 93019C |
