# Supplementary material for: Effectiveness and safety of rivaroxaban versus warfarin in Taiwanese patients with end-stage renal disease and nonvalvular atrial fibrillation: A real-world nationwide cohort study
Source: PLoS One. 2021 Apr 8;16(4):e0249940. doi: 10.1371/journal.pone.0249940 (PMC8031437; doi:10.1371/journal.pone.0249940)
Supplement: Attachment — Submitted filename: PLOS ONE review Comment to Author.docx [file pone.0249940.s003.docx]

Comments to the Author

Yi-Cheng Lin et al. examined the efficacy and safety of rivaroxaban and warfarin in patients with end-stage renal disease in a population-based cohort study.

The author has clearly shown valuable information that will change future clinical practice. I think this paper is a well-written and informative study.

Major comments

This paper is an important study analyzed in detail by population-based cohort study using National Health Insurance.

If possible, please consider the content of the following papers.

Biol Pharm Bull 2011;34:824-30: The role of PAR2 in the progression of renal function.(This paper is related to the other fact that type Xa DOAC prevents the role of PAR2)

In addition, as shown in Reference paper 20, comparing with antiplatelet drugs and examining dialysis cases leads to deeper consideration..
